# Magnetic resonance elastography (MRE) shows significant reduction of thigh muscle stiffness in healthy older adults

**DOI:** 10.1007/s11357-019-00147-2

**Published:** 2019-12-21

**Authors:** Paul Kennedy, Eric Barnhill, Calum Gray, Colin Brown, Edwin J.R. van Beek, Neil Roberts, Carolyn Anne Greig

**Affiliations:** 1 Edinburgh Imaging facility QMRI, School of Clinical Sciences, The University of Edinburgh, Edinburgh, EH16 4TJ UK; 2 BioMedical Engineering and Imaging Institute, Icahn School of Medicine at Mount Sinai, 1470 Madison Avenue, New York, NY 10029 USA; 3 Department of Radiology, Charité Universitätsmedizin Berlin, Berlin, Germany; 4 The Mentholatum Company Ltd., East Kilbride, Glasgow, UK; 5 School of Sport, Exercise and Rehabilitation Sciences, MRC-Arthritis Research UK Centre for Musculoskeletal Ageing Research, NIHR Birmingham BRC, The University of Birmingham, B15 2TT, Birmingham, UK

**Keywords:** Elastography, Ageing, Muscle, Stiffness

## Abstract

Determining the effect of ageing on thigh muscle stiffness using magnetic resonance elastography (MRE) and investigate whether fat fraction and muscle cross-sectional area (CSA) are related to stiffness. Six healthy older adults in their eighth and ninth decade and eight healthy young men were recruited and underwent a 3 T MRI protocol including MRE and Dixon fat fraction imaging. Muscle stiffness, fat fraction and muscle CSA were calculated in ROIs corresponding to the four quadriceps muscles (i.e. vastus lateralis (VL), vastus medialis (VM), vastus intermedius (VI), rectus femoris (RF)), combined quadriceps, combined hamstrings and adductors and whole thigh. Muscle stiffness was significantly reduced (*p* < 0.05) in the older group in all measured ROIs except the VI (*p* = 0.573) and RF (*p* = 0.081). Similarly, mean fat fraction was significantly increased (*p* < 0.05) in the older group over all ROIs with the exception of the VI (*p* = 0.059) and VL muscle groups (*p* = 0.142). Muscle CSA was significantly reduced in older participants in the VM (*p* = 0.003) and the combined quadriceps (*p* = 0.001), hamstrings and adductors (*p* = 0.008) and whole thigh (*p* = 0.003). Over the whole thigh, stiffness was significantly negatively correlated with fat fraction (*r* = − 0.560, *p* = 0.037) and positively correlated with CSA (*r* = 0.749, *p* = 0.002). Stepwise regression analysis revealed that age was the most significant predictor of muscle stiffness (*p* = 0.001). These results suggest that muscle stiffness is significantly decreased in healthy older adults. Muscle fat fraction and muscle CSA are also significantly changed in older adults; however, age is the most significant predictor of muscle stiffness.

## Introduction

Age-related effects on skeletal muscle include loss of muscle mass and decline in muscle force production, defined as sarcopenia, (Cruz-Jentoft et al. 2010) and increased fat accumulation within the muscle (Marcus et al. 2010). However, the change in muscle stiffness with age is less well described. Radiographic measurement of muscle stiffness includes contributions from muscle fibres, inter- and intramuscular fat and extracellular matrix (ECM) which supports the fibre bundles. Changes in each of these constituent parts may affect the overall stiffness measurement of the muscle. For example, the effect of ageing on muscle mechanical properties has been studied in ex vivo animal models (Wood et al. 2014) and revealed significantly increased extracellular matrix (ECM) stiffness in older rats. The ECM stiffness increase was interpreted to be due to accumulation of collagen and an increase in the concentration of advanced glycation end products (AGEs). AGEs are modified collagen molecules which form permanent tissue cross-links that stiffen collagen fibrils and which accumulate with age in slower turnover type I collagen. Age-related accumulation of AGEs has also been reported in older human skeletal muscle (Haus et al. 2007). Despite the increased ECM stiffness, Wood et al. reported no increase in muscle fibre stiffness suggesting that increased ECM stiffness was the cause for elevated muscle stiffness measures.

In the present study, we investigated the use of MR elastography (MRE) to noninvasively measure muscle mechanical properties, in young (< 29 years) and older adults (> 79 years). MRE is used to measure tissue stiffness by encoding displacements due to the propagation of externally induced acoustic waves into the MR phase signal. The speed of wave propagation depends on the stiffness of the underlying tissue, with waves travelling faster in stiffer tissues. MRE has been established as one of the most accurate methods of staging liver fibrosis (Kennedy et al. 2018); however it has also been employed as a means of measuring muscle mechanical changes under conditions such as contraction (Barnhill et al. 2013), injury (Kennedy et al. 2017) and in diseases such as Duchenne muscular dystrophy (Bensamoun et al. 2015). MRE enables simultaneous cross-sectional analysis of muscle groups including deep regions close to bone, which is difficult to achieve using ultrasound elastography. MRE studies of age-related change in muscle stiffness are limited, and there are some differences in the findings. For example, whereas Debernard et al. (Debernard et al. 2011) reported an increase in muscle stiffness between children and adults, Domire et al.*,* (Domire et al. 2009) found no significant difference in tissue stiffness between a group of combined young and middle-aged adults compared with a group of older adults. Domire et al. did, however, report that the highest stiffness measurements (i.e. shear modulus) were from the older participants. The authors also reported that standard deviation of tissue stiffness increased with age and suggested this could be a marker of increased heterogeneity of older muscles.

While age-related accumulation of AGEs may cause an increase in radiographic measurement of muscle stiffness, elevated proportions of adipose tissue within the muscle may precipitate a competing decrease in muscle stiffness. Fatty infiltration of muscle increases with advancing age (Marcus et al. 2010), with some studies suggesting a link between increased intermuscular fat and poor muscle function and performance (Tuttle et al. 2012). A study of healthy controls and patients with Duchenne’s muscular dystrophy found subcutaneous adipose tissue is less stiff than muscle tissue in healthy subjects (Bensamoun et al. 2015). This was confirmed in a study of muscle stiffness in healthy volunteers (Chakouch et al. 2015). Thus, increased intermuscular and intramuscular adipose tissue deposition may cause a reduction in the measured muscle stiffness. In this study, the Dixon imaging technique (Dixon 1984) is used to quantify muscle fat fraction. Previously Dixon imaging has been used to demonstrate increased fat fraction in older muscle (Azzabou et al. 2015). The method utilizes the inherent chemical shift present between water and fat. Manipulation of the imaging sequence echo time allows the acquisition of images with fat and water signals In-phase (IP) and out-of-phase (OP) and post-processing of the data enables quantification of the water and fat components within the image, generating a fat fraction map.

Whether or not an ageing muscle becomes more or less stiff compared with a younger muscle, a change in muscle stiffness could impact upon muscle mechanical behaviour by modifying the length-tension relationship and thus the force generating capacity of the muscle. This has been reported with respect to the ageing tendon, which is less stiff compared with younger tendon (Narici and Maffulli 2010). If the overall result was to be a decrement in force-generating capacity in a muscle already weakened due to age-related decreases in contractile tissue mass, the ability to perform everyday tasks important for the maintenance of physical independence could be compromised. Thus it is important to investigate the mechanisms underlying muscle weakness in older age in order to inform effective counteractive interventions. MRE is noninvasive and does not entail vigorous muscle activity, which is often used to determine muscle mechanical function; thus it may be particularly advantageous in studies of older adults with frailty.

The main objective of the present study was to determine whether muscle stiffness measured using MRE differed between healthy adults aged 79 years and older compared with young adults. Based on previous studies, we hypothesised that muscle stiffness would be increased in older age due to increased stiffness of connective tissue. A second objective was to measure muscle fat fraction and CSA to further characterize the participants and gain an insight into the relationship between fat accumulation, muscle size and mechanical properties.

## Methods

### Participants

The study was approved by the local Research Ethics Committee (REC) and conformed to the standards set by the Declaration of Helsinki. Informed written consent was obtained from fourteen men and women including eight healthy young men in their twenties (median age 23 years, range 22–29 years; BMI 25.6 ± 2.3) and six older adults (M/F 4/2, median age 83.5 years, range 79–87 years, BMI 23.8 ± 3.3). All participants were classified as healthy according to responses to a health questionnaire (Greig et al. 1994).

### MRI data acquisition

**Fig. 1:**
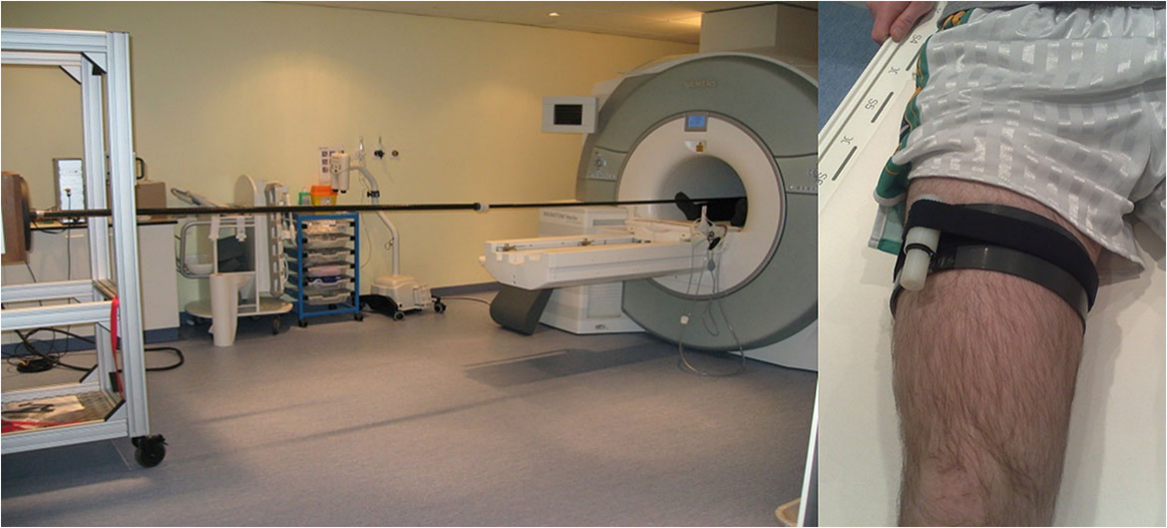
MRE apparatus including stationary loudspeaker and carbon fibre transmission rod which connected snugly to a plastic ring encompassing the thigh. Velcro straps ensured a tight fit to maximize wave transmission

Due to their importance in performing a wide range of physical activities in everyday life, the thigh muscles were the focus of study (Hurley et al. 1998; Moxley Scarborough et al. 1999). All measurements were obtained using a 3 T MRI system (MAGNETOM Verio, Siemens AG). Participants lay supine with the midpoint of the right femur positioned at magnet isocentre and marked with a cod liver oil capsule. Mechanical excitation was introduced to the muscle via a plastic ring positioned 2 cm below the mid-femur point and which was connected to the vibration source via a carbon fibre piston. The ring was firmly secured to the leg using Velcro strapping to ensure good wave transmission (Fig. 1).

**Fig. 2:**
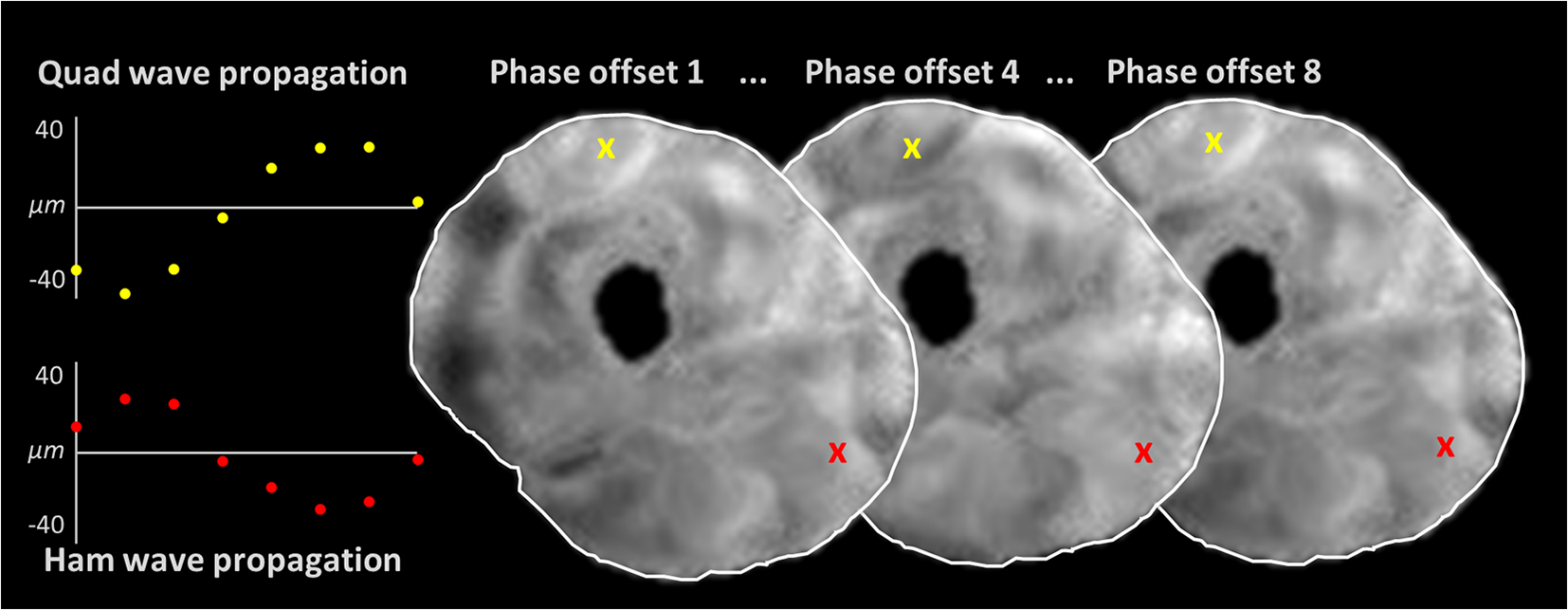
Illustrative example of through-plane wave propagation captured over 8 phase offsets. Displacement plots depict the wave propagation in an example pixel in the quadriceps (yellow marker) and hamstrings (red marker) over each phase offset

Localiser scans were performed prior to MRE to identify the position of the mid-femur landmark. MRE data were acquired using a modified single-shot EPI sequence, sensitized to motion perpendicular to the image plane. Imaging parameters were TR = 1600 ms, TE = 56 ms, FOV = 235 mm, matrix = 128 × 128, slice thickness = 10 mm, scan duration = 40 s, 8 phase offsets acquired capturing the wave propagation (Fig. 2). The external vibration frequency was 50 Hz, with a matching 50 Hz motion-encoding gradient. A three-point Dixon sequence (T1-weighted spoiled gradient recalled (SPGR)) with echo times of 2.46 ms and 8.61 ms (corresponding to the OP and IP times of water and fat MR signals) was used to calculate fat fraction and for muscle CSA measurement. Images were also acquired with an additional OP echo (TE = 4.92 ms) to enable calculation of a T2* correction value. Other parameters were TR = 213 ms, FA = 70̊, FOV = 460 mm, acquisition matrix 256 × 256, and slice thickness = 8 mm.

### MRE data analysis

**Fig. 3:**
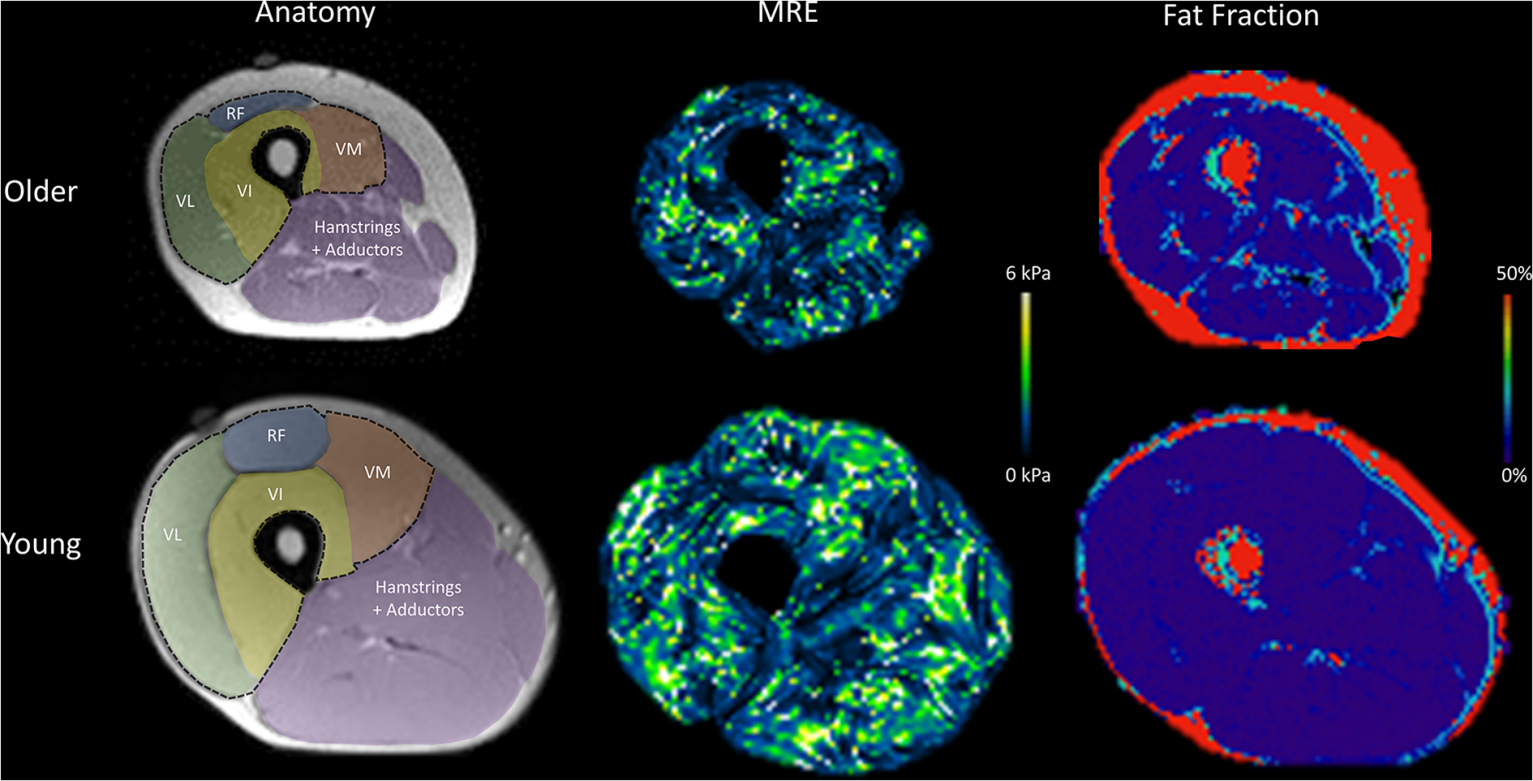
Example anatomy, MRE and fat fraction images from an older and young participant. Cod liver oil capsule denoting the mid-femur point is visible on the anterior surface of the thigh. ROI regions are overlaid on the anatomical image. Quadriceps ROI is represented by black dotted line incorporating RF, VI, VL and VM muscle groups. Whole-thigh ROI includes both quadriceps and hamstrings and adductors ROI. Fat fraction pixel values are scaled from 0 to 50% to aid visualization

The assumption that a ring-shaped actuator will generate planar waves, which are necessary for 2D MRE (Bishop et al. 2000), has previously been shown to be valid (Klatt et al. 2010), and additionally assumptions of muscle as a linearly viscoelastic, incompressible, locally homogeneous solid (Manduca et al. 2001) were applied. Phase accumulations of greater than ± 2π were corrected using a Laplacian-based phase unwrapping algorithm (Barnhill et al. 2015). The data were then imported to MATLAB, and a custom pipeline (Barnhill et al. 2016) was used to calculate the magnitude of the complex modulus, |G*|. Regions of interest (ROIs) corresponding to the four main quadriceps muscles (i.e. vastus lateralis (VL), rectus femoris (RF), vastus intermedius (VI), vastus medialis (VM)), combined quadriceps muscle, combined hamstrings and adductors and whole thigh (quadriceps, hamstrings and adductors) were manually segmented using ImageJ software (Fig. 3). In areas bordering subcutaneous fat, ROIs were drawn conservatively to reduce the contribution from partial volume effects.

### Fat fraction and muscle CSA analysis

Fat fraction was calculated from the signal intensity of the OP (TE 2.46 ms and 4.92 ms) and IP (TE = 8.61 ms) images as described in (Lee and Yu 2014) for the same ROIs as MRE. CSA was also calculated for the same individual muscle groups, quadriceps, hamstrings and adductors and whole thigh as the MRE and fat fraction measurements. The cross-sectional area of the muscle was quantified on each image by drawing a region of interest around the muscle using ImageJ software. For CSA determination, the muscle was traced accurately around the whole perimeter including in areas adjacent to subcutaneous fat. Classification of sarcopenia status of the whole thigh of the older muscles was based on muscle size criteria (Janssen et al. 2002).

### Statistical analysis

All data are presented as mean ± standard deviation. Differences in muscle stiffness, fat fraction and CSA between groups were tested for significance using Mann-Whitney U tests. Stepwise multiple regression was used for multivariate analysis of factors potentially affecting muscle stiffness. A two-tailed *p* value < 0.05 was considered significant. All statistical analyses were performed using SPSS (Version 20, IBM).

## Results

The MRI scanning session lasted approximately 30 min and was well tolerated by all participants with no adverse events. Example anatomical, MRE and fat fraction images obtained from a young and older participant are shown in Fig. 3.

**Fig. 4:**
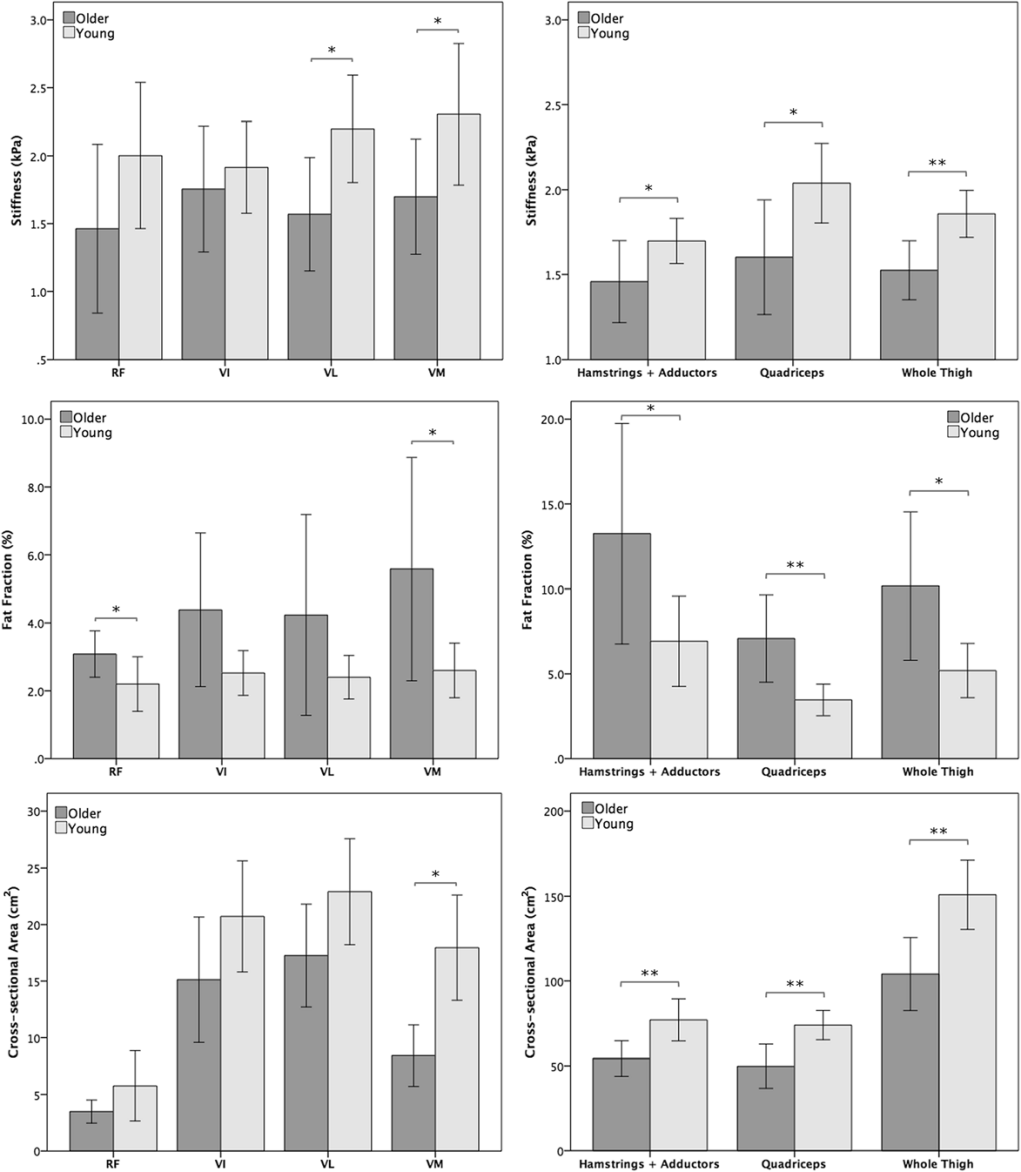
Bar charts representing (**a**) |G*|from older and young participants in individual muscle groups (left) and combined ROIs (right), (**b**) fat fraction in older and young participants in individual muscle groups (left) and combined ROIs (right), (**c**) muscle CSA from older and young participants in individual muscle groups (left) and combined ROIs (right). *signifies *p* < 0.05. **signifies *p* < 0.01

MRE measurements of magnitude of the complex modulus, |G*|, indicated lower mean stiffness in all ROIs in the older group compared to the young group (Table 1), of which a significant difference was found in VL (1.57 ± 0.42 kPa vs 2.20 ± 0.39 kPa, *p = 0.02*) and VM (1.70 ± 0.43 kPa vs 2.30 ± 0.52 kPa, *p = 0.043*) muscle groups, quadriceps (1.60 ± 0.34 kPa versus 2.04 ± 0.23 kPa, *p = 0.043*), combined hamstrings and adductors (1.46 ± 0.24 kPa versus 1.70 ± 0.13 kPa, *p = 0.043*) and whole thigh (1.52 ± 0.17 kPa versus 1.86 ± 0.14 kPa, *p = 0.005*, Fig. 4a).

**Table 1:** Descriptive statistics of stiffness, fat fraction and cross-section area in individual muscles and muscle groups in 14 subjects

	Stiffness (kPA)			Fat fraction (%)			Cross-sectional area (*cm*^2^)		
	Older	Young	*ρ*	Older	Young	*ρ*	Older	Young	*ρ*
RF	1.46 0.62	2.00 ± 0.54	0.081	3.1 ± 0.7	2.2 ± 0.8	**0.043**	3.49 ± 1.02	5.76 ± 3.10	0.142
VI	1.76 ± 0.46	1.91 ± 0.34	0.573	4.4 ± 2.3	2.5 ± 0.7	0.059	15.12 ± 5.54	20.72 ± 4.92	0.108
VL	1.57 ± 0.42	2.20 ± 0.39	**0.02**	4.2 ± 3.0	2.4 ± 0.6	0.142	17.26 ± 4.55	22.91 ± 4.69	0.059
VM	1.70 ± 0.43	2.30 ± 0.52	**0.043**	5.6 ± 3.3	2.6 ± 0.8	**0.029**	8.42 ± 2.71	17.96 ± 4.66	**0.003**
Quadriceps	1.60 ± 0.34	2.04 ± 0.23	**0.043**	7.1 ± 2.6	3.5 ± 0.9	**0.003**	49.76 ± 12.99	73.91 ± 8.62	**0.001**
Hamstrings	1.46 ± 0.24	1.70 ± 0.13	**0.043**	13.3 ± 6.5	6.9 ± 2.7	**0.029**	54.28 ± 10.41	76.97 ± 12.42	**0.008**
Whole Thigh	1.52 ± 0.17	1.86 ± 0.14	**0.005**	10.2 ± 4.4	5.2 ± 1.6	**0.013**	104.03 ± 21.55	150.88 ± 20.46	**0.003**

Mean ± SD of stiffness, fat fraction and CSA measurements over all ROIs. *p* value from Mann-Whitney U-tests are also included. *RF* rectus femoris, *VI* vastus intermedius, *VL* vastus lateralis, *VM* vastus medialis

Mean fat fraction was increased over all ROIs in the older group (Table 1), with a significant increase in the RF (3.1 ± 0.7% vs 2.2 ± 0.8%, *p* = 0.043) and VM (5.6 ± 3.3% vs 2.6 ± 0.8%, *p* = 0.029) muscle groups and combined quadriceps (7.1 ± 2.6% vs 3.5 ± 0.9%, *p* = 0.003), hamstrings and adductors (13.3 ± 6.5% vs 6.9 ± 2.7%, *p* = 0.029) and whole thigh (10.2 ± 4.4% vs 5.2 ± 1.6%, *p* = 0.013, Fig. 4b).

Muscle CSA showed a similar direction of change to the stiffness measurements, in that mean CSA was lower in the older compared to the younger group over all measured ROIs (Table 1). The reduction was significant for VM (8.42 ± 2.71 vs 17.96 ± 4.66 cm^2^, *p* = 0.003), quadriceps (49.76 ± 12.99 vs 73.91 ± 8.62 cm^2^, *p* = 0.001), hamstrings and adductors (54.28 ± 10.41 vs 76.97 ± 12.42 cm^2^, *p* = 0.008) and over the whole thigh (104.03 ± 21.55 vs 150.88 ± 20.46 cm^2^, *p* = 0.003, Fig. 4c). Based on CSA of the whole thigh, all the older participants were sarcopenic with 3/6 (50%) of the older adult group fulfilling the criteria for class II sarcopenia (i.e. < 2 SD below mean CSA of the young adults) and the remaining participants exhibiting class I sarcopenia (i.e. < 1 SD below mean CSA of the young adults) (Janssen et al. 2002).

**Fig. 5:**
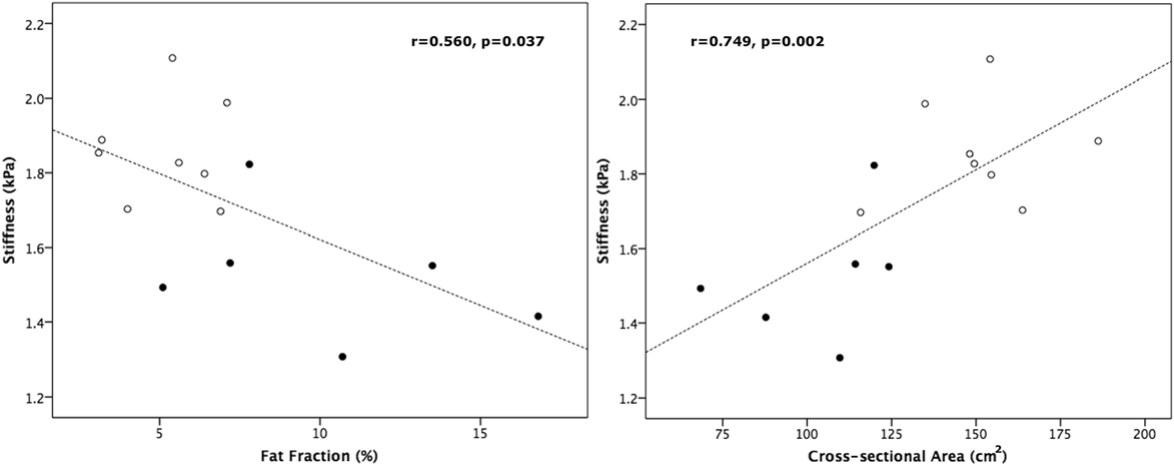
Scatterplots showing the correlation between stiffness and fat fraction (left), and stiffness and CSA (right) over the whole thigh. Filled data points represent the older participants

Due to low participant numbers, correlations were only assessed over the largest ROI, the whole thigh (Fig. 5). MRE was found to significantly negatively correlate with fat fraction (*r* = − 0.560, *p* = 0.037) and positively correlate with CSA (*r* = 0.749, *p* = 0.002). Stepwise regression analysis was performed with whole-thigh MRE as the dependent variable and age, whole-thigh fat fraction and whole-thigh CSA as independent predictors. The resultant model revealed that age was the most significant predictor of muscle stiffness (*p* = 0.001).

## Discussion

The results of this study indicate that in older participants, who are all over 79 years of age, muscle stiffness is significantly reduced compared to young adults in their twenties. Moreover, fat fraction was significantly increased, and muscle CSA was found to be significantly reduced in older adults. Stepwise regression analysis found age to be the most significant predictor of muscle stiffness.

MRE has been used previously to measure age-related changes in skeletal muscle (Debernard et al. 2011; Domire et al. 2009), but this is the first study to report MRE-derived muscle stiffness obtained from healthy men and women in their eighth and ninth decades with lower muscle mass and higher fat fraction than their younger counterparts. Previous MRE studies have found no significant effect of age on stiffness, although the highest measured stiffness measurements were from older participants in one study (Domire et al. 2009). In the same study, an increased standard deviation was reported in older participants and attributed to increased tissue heterogeneity. In the current study, we did not replicate this result, although the disparity in the MRE analysis pipelines used in both studies renders this analysis difficult to compare owing to different filtering methods and smoothing filters applied during reconstruction. The acquisition methodology also differed, with the vibration frequency (50 Hz vs 100 Hz) and image plane (axial vs sagittal) potentially introducing further variability.

The effect of ageing on muscle tissue stiffness has been investigated by several groups using ultrasound elastography (USE). In one study (Akagi et al. 2015), muscle shear modulus was found to be significantly lower in rectus femoris, gastrocnemius and soleus in participants aged 65 years and older compared with younger participants. Another USE study found that resting muscle stiffness of rectus femoris was lower in older adults (*N* = 10, mean age 57 years) compared with a younger group (*N* = 10, mean age 27 years), but the difference did not reach statistical significance (Wang et al. 2014). In contrast, a study using USE to measure the stiffness of the biceps brachii muscle of the upper arm at rest in men and women aged 21–94 years reported increases in muscle stiffness with increasing age (Eby et al. 2015), a finding which is not supported by the results of the present study. However, upon closer inspection of the data most relevant to the ages of the participants in the present study (i.e. ages 20 to 29 years and 80 to 89 years), some consistency is observed which was previously obscured by the respective sex distributions of participants within each study. In particular, although mean combined muscle stiffness for the 80–89-year-old men and women in Eby et al. was higher compared with that of the 20–29-year-old group, the muscle stiffness of the 80–89-year-old men was lower than the 20–29-year-old men (4.24 ± 0.67 kPa vs 5.32 ± 2.38 kPa at 90° flexion, 12.81 ± 2.03 kPa vs 15.52 ± 5.07 kPa at full extension) a mean stiffness decrease of ~ 20%. In the present study, the older group comprised mainly men (4 men and 2 women), and they had a similarly reduced stiffness over whole thigh (22%) compared with the younger group.

The effect of gender on muscle stiffness, both in younger and older subjects, has been the subject of some research but has not reached consensus. In the aforementioned study by Eby et al., the authors noted an increased biceps brachii muscle stiffness in women compared to men across all ages. An earlier USE study reported increased mean gastrocnemius and masseter muscle stiffness in men compared to women but found no significant difference (Arda et al. 2011). A recent study (Saeki et al. 2019) using torque-angle curves to assess muscle stiffness found higher soleus stiffness in young women compared to men but no significant difference in gastrocnemius stiffness. These discrepant studies suggest that gender differences may be dependent on the muscle studied and the technique employed to do so. Unfortunately the small sample size and lack of a balanced sex distribution mean we cannot speculate on sex differences within our data.

Increased stiffness in the calf muscles of older women has been reported (Gajdosik et al. 2005); however, the stiffness measurements were obtained by biopsy of muscle tendon units, which inherently contain contributions from articular structures not present in the muscle belly. The results of the current study could suggest an alternative mechanism counteracting the effects of stiffening ECM in older age. Quadriceps and whole-thigh fat fraction were significantly increased in the older compared with the young group. This confirms data recently published which showed increased thigh fat fraction in participants in their 60’s compared to participants in their 30’s (Yoon et al. 2017). Fat tissue is inherently less stiff compared with muscle tissue (Bensamoun et al. 2015; Chakouch et al. 2015) and therefore increased fat infiltration may potentially lead to a decrease in overall stiffness.

MRI is a well-established technique for visualization and estimation of muscle CSA. In the present study, the CSA of the VM muscle group, quadriceps, combined hamstrings and adductors and whole thigh were significantly reduced in the older group compared with the young group, with the older group fulfilling the criteria for class I or II sarcopenia over the thigh cross-section. This finding supports results obtained from a number of groups (Gray et al. 2011), that even in good health, older muscles are smaller than younger muscles. Further research is required to establish if this relationship holds in older subjects who are not sarcopenic and to determine the effect of muscle size on measured stiffness.

Our measurements were made with the participant’s muscles in a passive state, and the effect of increased fat fraction and reduced CSA on contractile response was not investigated. An earlier study of the stiffness of muscle measured with a myotonometer in young and older women (Ikezoe et al. 2012) found that during contraction, muscle stiffness was significantly reduced in older compared with younger women. A slightly higher mean resting stiffness was reported in the older group, but the difference was not significant. In the same study the authors estimated fat infiltration in the muscles via ultrasound echo intensity of the muscle and found a significantly increased echo intensity in the older group, suggestive of increased fat infiltration.

 addition to not assessing muscle contractile response, in this study, participant’s physical activity was not recorded. All of the older participants were mobile and did not use walking aids or wheelchairs. Anecdotally we learned that some members of the younger group engaged in casual sporting activity whilst others were sedentary. Previous studies have identified an association between muscle mass and physical activity (Proctor et al. 2000). In our study, muscle mass was not determined however whole thigh CSA ranged from 6.84–12.41 cm^2^ in the older group and 11.58–18.61 cm^2^ in the younger group. Although there was a significant difference in CSA, there was some overlap suggesting a moderately active older group. The relationship between muscle stiffness, muscle strength and physical activity is complex, with some one study not finding an association between muscle strength and physical activity (Hughes et al. 2001). Further research including functional assessment in addition to radiological measurements such as MRE is required.

The methods used here have been validated in previous work (Barnhill et al. 2013; Klatt et al. 2010). However, this study had several limitations. In particular, data analysis for MRE is almost always performed under the assumption that the tissue is homogenous and isotropic (Manduca et al. 2001). However, muscle tissue is inherently anisotropic, and more detailed analysis using anisotropic inversion methodologies have been developed. One approach is to use diffusion tensor imaging (DTI) (Green et al. 2013) to determine the structural symmetry axis of the muscle fibres and incorporate this information in the numerical solution of the wave equation (Bilston and Tan 2014). Methodologies based on a model in which muscle fibres are isotropic with respect to the transverse plane of the muscle have also been proposed (Guo et al. 2016). The use of 2D MRE rather than 3D MRE precluded the use of these advanced analysis methods in this study. 3D MRE data acquisition samples the propagating shear waves in all 3 directions rather than only the through plane direction as used in 2D MRE. The richer dataset captured with 3D MRE may address issues inherent with 2D MRE such as elevated stiffness measurement in cases of oblique wave propagation through the imaging plane. 3D MRE is currently a research application and was not available on the MR system used in this study during data acquisition. Additionally, although every effort was made to ensure good contact between the cuff and the participant’s leg, the leg diameter of several older participants was so small that additional support was required to keep the plastic cuff in contact with the thigh. Finally, an additional limitation was that the extent to which participants regularly engaged in physical activity was not recorded, which may potentially confound the interpretation of muscle ageing effects.

In conclusion, an MRI protocol incorporating measurements of muscle stiffness, fat fraction and CSA has revealed that muscle stiffness is significantly reduced in older adults compared with young adults. The older adults also exhibited greater fat infiltration and smaller muscle size. This research should be considered preliminary due to the small number of participants. However, the significant finding of decreased muscle stiffness in older adults compared with young adults warrants further study. In particular, an analysis of muscle stiffness over a wider age range would be informative in terms of identifying whether there is a ‘threshold’ age at which muscle stiffness may begin to decline. This type of analysis would be strengthened by inclusion of functional measures (such as chair rise or walking speed) in order to explore whether any decline in stiffness is associated with changes in the ability to undertake tasks and activities important for the maintenance of physical independence. The increased presence of fat, with an inherent lower stiffness compared to muscle, appears to cause stiffness measurements of upper-leg muscles to be lower than expected, and this may be further exacerbated by a reduced number of muscle fibres in older muscles causing an exaggerated effect. Further MRE muscle research of larger cohorts, with a wide age range and in whom physical activity levels are known, is needed to determine the reproducibility and further inform the interpretation of the present findings.

## Abbreviations

AGE: Advanced glycation end products
CSA: Cross-sectional area
ECM: Extracellular matrix
MRE: Magnetic resonance elastography
RF: Rectus femoris
USE: Ultrasound elastography
VI: Vastus intermedius
VL: Vastus lateralis
VM: Vastus medialis
